# Humic Substances as Microalgal Biostimulants—Implications for Microalgal Biotechnology

**DOI:** 10.3390/md20050327

**Published:** 2022-05-16

**Authors:** Daria Gabriela Popa, Carmen Lupu, Diana Constantinescu-Aruxandei, Florin Oancea

**Affiliations:** 1 Faculty of Biotechnologies, University of Agronomic Sciences and Veterinary Medicine of Bucharest, Mărăști Blv, No. 59, Sector 1, 011464 Bucharest, Romania; daria.popa@icechim.ro; 2 Bioproducts Team, Bioresources Department, National Institute for Research & Development in Chemistry and Petrochemistry—ICECHIM, Splaiul Independenței No. 202, Sector 6, 060021 Bucharest, Romania; carmen.lupu@icechim.ro

**Keywords:** humic substances, microalgae cultivation, hormetic effects, increased nutrient availability, improved protection against abiotic stress, higher accumulation of bioactive ingredients, enhanced microalgal productivity

## Abstract

Humic substances (HS) act as biostimulants for terrestrial photosynthetic organisms. Their effects on plants are related to specific HS features: pH and redox buffering activities, (pseudo)emulsifying and surfactant characteristics, capacity to bind metallic ions and to encapsulate labile hydrophobic molecules, ability to adsorb to the wall structures of cells. The specific properties of HS result from the complexity of their supramolecular structure. This structure is more dynamic in aqueous solutions/suspensions than in soil, which enhances the specific characteristics of HS. Therefore, HS effects on microalgae are more pronounced than on terrestrial plants. The reported HS effects on microalgae include increased ionic nutrient availability, improved protection against abiotic stress, including against various chemical pollutants and ionic species of potentially toxic elements, higher accumulation of value-added ingredients, and enhanced bio-flocculation. These HS effects are similar to those on terrestrial plants and could be considered microalgal biostimulant effects. Such biostimulant effects are underutilized in current microalgal biotechnology. This review presents knowledge related to interactions between microalgae and humic substances and analyzes the potential of HS to enhance the productivity and profitability of microalgal biotechnology.

## 1. Introduction

Humic substances (HS) are a significant part of soil organic matter. HS are considered important for soil fertility due to their ability to retain water and nutrients, to improve soil cation exchange capacity (CEC), to increase nutrient availability and to generate aerated soil structure [1,2,3]. Soil scientists separate HS into humic acids (HA), soluble at alkaline pH and insoluble at acidic pH, and fulvic acids (FA), which are soluble both at alkaline and acidic pH [4,5].

HS are supramolecular structures resulting from the association of small molecules derived from slow degradation of biological material, especially plant residues, under specific conditions [6,7]. The supramolecular structure of HS comprises hydrophilic portions, including -OH and -COOH groups, and hydrophobic portions [6,8]. The hydrophobic portions are represented mainly by networks of polyaromatic hydrocarbons derived from lignin [9,10]. The following organic molecules are linked to this hydrophobic scaffold: organic acids, mainly derived from fermentative carbohydrate metabolism [11]; substances derived from protein metabolism, including amino acids [12,13,14] and polyamines [15]; aliphatic hydrocarbons, which result from waxes; and cross-linked fatty acids [16]. FA are more polar than HA, containing smaller amounts of hydrophobic fragments and larger quantities of organic (poly)acids than HA [17]. Humic acids have a more pronounced amphiphilic structure than FA [18]. Such structures promote aggregation in the supramolecular structures stabilized by hydrophobic interactions [19].

The genesis of humic substances in the soil is still under dispute [20]. The majority consider that HS result from polymerization and polycondensation of the components generated in soil by the decomposition of plant residues and soil microorganisms [21,22,23]. Such (medium) molecular weight structures are further aggregated into supramolecular structures, according to their polarity index and/or hydrophobic/hydrophilic ratio [24,25], in a reaction that is catalyzed by soil microorganisms [26,27,28] or soil inorganic materials [27,29]. Another opinion considers HS generation as being the result of the “selective preservation” and “progressive decomposition” of biological material in soils [1].

Despite the controversies related to their formation, the HS-specific structure determines the HS-specific features. The phenolic and carboxylic groups are responsible for the HS weak acid behavior and pH buffering [30]. The quinones–phenols switch is involved in redox buffering activity [25,31]. The coexistence of the hydrophobic and hydrophilic portions in HS supramolecular structure determines several properties of HS, such as the (pseudo)emulsifier effect and tendency to form micelles [32,33] and potential encapsulation of labile hydrophobic compounds in the hydrophobic pocket [34]. Such properties are related to the HS effects on biological systems. For example, the HS reactivity toward different (micro)biocenosis components is related to the ratio between hydrophilic and hydrophobic components [35,36].

HS are plant biostimulants, promoting nutrient uptake and nutrient use efficiency. Root dry weight increases by more than 20% after exogenous HS application [37]. A large body of evidence demonstrated the auxin-like activity of the humic and fulvic acids, including the rhizogenesis and induction of the proton-pump H^+^-ATPase [38]. Activation of the secondary ion transport occurs after the change of radicular cell membrane potential [39]. An increase in ionic nutrient uptake following HS application was demonstrated for nitrate [40,41], phosphate [42], and nitrate and sulfate [43]. Ionic nutrient uptake is also stimulated due to enhanced nutrient availability resulting from the chelating effect of humic substances [44], combined with their redox properties [3]. HS also promote primary anabolic pathways, such as nitrate reduction [45] and carbon catabolism [46], leading to enhanced nutrient use efficiency.

Transcriptomic and metabolomic analyses reveal significant modifications of metabolic pathways in both dicotyledonous and monocotyledonous plants under HA treatments. The HA extracted from black peat in alkaline conditions and separated from fulvic acids by HCl precipitation activated the carbon (C)-, nitrogen (N)- and sulfur (S)- metabolic pathways in rapeseed seedlings (*Brassica napus* var. Capitol) [43]. In sugarcane, *Saccharum officinarum* cv. RB 96 7515, application of HA extracted from vermicompost activated the metabolic pathways related to plant stress response and cellular growth [47]. In corn, *Zea mays* cv. PAN 3Q-240, soil application of HS formulation determined several effects according to the nutrient status of the plant. In plants supplied with normal quantities of mineral nutrients, HS stimulated the metabolic pathways related to primary metabolisms—tricarboxylic acid (TCA) cycle and amino acid metabolism. In plants under mineral nutrient deficit, HS stimulated the secondary metabolism pathways related to stress response [48].

Induction of H^+^-ATP-ase in root cells by HS elicits the internal cell signals, including an increased level of nitric oxide (NO) [49] and calcium ion influx, followed by the activation of the calcium-dependent protein-kinase (CDPK) [50].

Humic substances activate plant secondary metabolism by (bio)chemical priming [51]. The mechanism of chemical priming by HS in plant tissues and cells involves an increased level of reactive oxygen species (ROS) and modulation of polyamine metabolism [44]. Activation of secondary metabolism determines higher plant tolerance to abiotic stress and increased accumulation of compounds resulting from the plant secondary metabolism in the edible yield [52].

The complex activity of HS on terrestrial plants is related to the complexity of the HS chemical systems. Complexity in its ”emergence” aspect is an essential characteristic of first-generation plant biostimulants [53]. The HS complex chemical system presents emergent features, i.e., unexpected properties resulting from component interaction. One illustrative example is the synergic interaction between hydrophobic and hydrophilic moieties in water-holding in arid soil, according to relative humidity. Another feature related to HS system complexity is the context-dependent characteristics, such as redox properties [54]. Due to this intrinsic complexity, combined methods are needed to characterize the HS used as plant biostimulants [55].

In contrast to terrestrial plants, studies related to HS effects on microalgae are relatively scarce. The initial driver of studying the HS effects on microalgae was an ecotoxicological one. Due to the growing interest in carbon sequestration by microalgae, HS has been proposed as a biostimulant for microalgae in recent years [56,57].

HS effects on microalgae are more pronounced than on terrestrial plants. In aqueous solutions/suspensions, HS generates a more dynamic supramolecular structure than in soil [58]. Due to the dynamic structure of HS in solution/suspension, the main HS features, such as pH and redox buffering, ionic nutrient complexation, encapsulation of hydrophobic molecules, and emulsifying characteristics are significantly enhanced [23]. The biological activities related to these HS characteristics are also enhanced. Therefore, the potential of HS to act as a biostimulant, including as support to enhance tolerance to various stresses, is higher in microalgae than in terrestrial photosynthetic organisms. At present, such potential is underutilized in microalgal biotechnology.

This paper reviews the interactions between HS and microalgae and analyzes the practical implications for microalgal biotechnology resulting from the specific effects of HS on microalgae.

## 2. Hormetic Effects of Humic Substances on Microalgae

As was already mentioned, the initial studies investigated the influence of HS leached from soil on phytoplankton. The positive effect of humic substances on phytoplankton growth was reported almost 50 years ago [59]. More recent studies demonstrated that higher doses of HS from various sources determined inhibition of microalgal growth. Such a dual response is typical of hormesis. Hormesis is a dose–response phenomenon characterized by low-dose stimulation and high-dose inhibition. Hormetic effects are typically graphed as an inverted U-shaped dose–response and a J-shaped dose–response, depending on the endpoint evaluated.

The microalgal response to humic substances is consistent at different cellular and biochemical levels. Various HA concentrations were used to treat *Scenedesmus capricornus* microalgae. At HA concentrations lower than 2.0 mg C L^−1^, the growth of *S. capricornus* was promoted slightly, and above 2.0 mg C L^−1^, it was inhibited [60]. The same study reported that an increase in polysaccharide content was observed at low HA concentrations (less than 2.0 mg C L^−1^), and that with an increment in HA concentration, it decreased. At 10.0 mg C L^−1^ HA, the average polysaccharide concentration was only 12.02 mg L^−1^ compared with the 18.43 mg L^−1^ average for the control group.

When studying the impact of a range of concentrations of HA (0.001–0.007%) isolated from six soil types on *Chlorella vulgaris* microalgae strains, the results illustrated an adverse effect of HA for all preparations at HA concentrations above 0.003%. At concentrations higher than 0.003%, the photosynthesis rate in *C. vulgaris* cells decreased, and respiration increased abruptly [61].

HS influenced the photosynthetic performance and stress response of two green algae, *Raphidocelis subcapitata*, strain 61.81 and *Monoraphidium braunii* strain 2006. These eukaryotic microalgae were exposed to four different concentrations of HS—0.17, 0.42, 1.67, and 4.17 mM dissolved organic carbon (DOC). The results highlighted that the dry weight per cell ratio decreased with increasing HS concentration. In contrast, the exposure to lower concentrations of HS stimulated better growth of the phototrophs and increased the quantum efficiency of photosystem II [62]. In the same paper, the authors reported a different response by the prokaryotic microalgae (cyanobacteria) *Synechocystis* sp. (PCC 6803) and *Microcystis aeruginosa* (PCC 7806). The tested *Synechocystis* strain was less sensitive to HS. The quantum efficiency of photosystem II was not increased in *M. aeruginosa* PCC 7806. However, in this cyanobacterial strain, the chlorophyll *a* content increased at the highest HS concentration tested compared to control.

The difference in response to HS by eukaryotic and prokaryotic microalgae species was demonstrated in an initial paper of the Steinberg group. The prokaryotic strain *Chroococcus minutus* 276-4b and the eukaryotic strain *Desmodesmus communis* 41.71 were compared. The authors considered that prokaryotic cells that lack internal membrane-delimited organelles would generally be more sensitive to HS than eukaryotic cells, with a cellular organization with several internal membrane-delimited compartments. When exposing the two species to HS at concentrations of 0.3 and 1.5 mg L^−1^ DOC, the authors reported an increased the number of cell of *D. communis* under low HS concentrations and an inhibitory effect at the highest concentrations. The cyanobacterium showed reduced photosynthetic activity and reduced population growth across the entire concentration range of HS tested [63].

HS extracted from lignite (Biomin) with 61.2% C composition had the same effect on eukaryotic (*Scenedesmus acutus* Meyen Tomaselli 8, *C. vulgaris* C-3) as compared to prokaryotic (*Anabaena variabilis* 786, *Nostoc commune*) microalgae strains. The microalgae cultures showed an average biomass increase of 18 and 15% compared to control for 1 and 10 mg L^−1^ Biomin, respectively. Negative effects of treatment with concentrations higher than 1 g L^−1^ HS were recorded, such as very significant decreases in protein, carbohydrate, and chlorophyll contents [64].

Papers presenting such dual/hormetic responses of microalgae to HS are summarized in Table 1.

**Figure 1:**
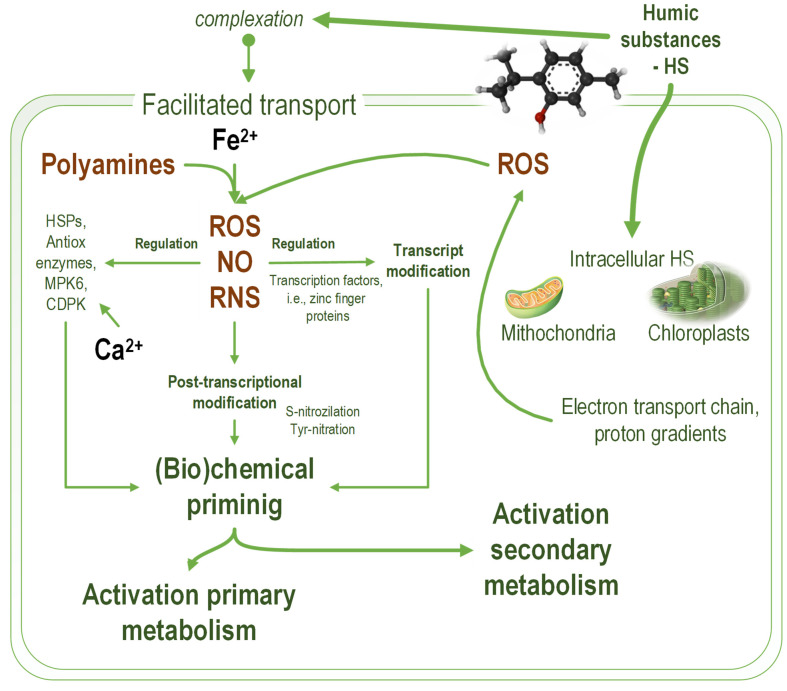
The mechanisms of humic substances (HS) effects on microalgae cells. HS increase membrane permeability for calcium and ferrous ions and diffuse through the plasmatic membrane. Ferrous ions promote the formation of reactive oxygen species (ROS) by redox reaction and activate the formation of nitric oxide (NO) from polyamines. Calcium ions activate specific protein kinases involved in cellular signaling. Intracellular HS interfere with the electron transport chain in chloroplasts and mitochondria, producing a higher level of reactive oxygen species. The simultaneous increase in NO and ROS levels causes an accumulation of reactive nitrogen species (RNS). The resulting nitrosative stress causes the development of physiological compensation mechanisms, which ultimately lead to the activation of primary and secondary metabolism. An increase in oxidative and nitrosative stress over the physiological thresholds damages cell function. HSPs—thermal shock proteins; MPK6—mitogenically activated protein kinases; CDPK—calcium-dependent protein kinases.

In the aquatic system, HS are a component of dissolved organic matter. HS molecules also represent a C and N source for microalgae at low concentrations. Additional beneficial effects of HS result from the overlapping effects of enhanced availability of nutrients and (bio)chemical priming (similar to terrestrial plants) generated by the higher physiological level of reactive oxygen and nitrogen species. At higher concentrations, the HS effects on microalgae are dominated by their interference with photosynthetic structures and the production of the pathophysiological level of reactive oxygen [65] and nitrogen species [66]. In Figure 1, the overlapping mechanisms of HS action in microalgae leading to stimulatory or inhibitory effects are detailed.

Modifying membrane permeability for various ionic species due to various HS effects, including complexation, generates elevated intracellular levels of redox-active ionic species, such as Fe^2+^, and ionic species acting as secondary messengers, e.g., Ca^2+^ [67,68]. The ability of HS to complex ionic species, especially iron ionic species, is important for the HS effect on microalgae [69]. An increased level of redox-active iron determines higher levels of reactive oxygen species [70,71]. The hydrophobic–hydrophilic ratio of humic substances is important for the effect on microalgae and the complexation of iron ionic species. The hydrophobic components determine high growth rates of *R. subcapitata* (synonym used *Pseudokirchneriella* *subcapitata*). The hydrophilic components inhibit microalgae growth due to reduced iron ionic species bioavailability [72]. The permeability change in the membrane of *R. subcapitata* depends on pH. The passive diffusion of fluorescent tracers through the membranes of *R. subcapitata* (synonym used *Selenastrum capricornutum*) is higher at pH 5 than at pH 7 [67].

Another HS mechanism of action is related to chloroplasts and mitochondria as a source of ROS involved in controlling the cellular response to stress factors [73]. In terrestrial plants, it was demonstrated that chloroplasts have an essential role in plant defense against stresses [74,75]. In plants, chloroplasts also dominate the primary metabolic pathways from plants—i.e., C-, N- and S-assimilation [76]. Therefore, their activation should determine an enhanced primary metabolism and, consequently, enhanced nutrient uptake and utilization. Activation of plant defense by chloroplast is associated with activation of the secondary metabolism through retrograde signaling [77]. In microalgae, the chloroplast retrograde signaling mechanism is similar to that described in plants because initially, such mechanisms evolved in the algal ancestors of the terrestrial plants [78]. ROS production by chloroplasts through photosystem II is involved in the microalgae intracellular communication network [79].

HS interactions with microalgae cells modulate the function of photosystem II. Due to their amphiphilic structure, humic substances diffuse through the microalgae cell membrane [18]. The intracellular humic substances modify chloroplast inner and outer membrane permeability [60]. At low concentrations, HS support the plastoquinone function as a trans-membrane proton shuttle, increasing the efficiency of photosystem II [62]. At higher concentrations, HS cause plastid homeostasis imbalance due to the destructuration of the proton gradient across the inner chloroplast membrane [80]. This proton gradient is fundamental for converting light energy into chemical energy (ATP) and is essential for the efficient functioning of photosystem II [81].

The complex nature of humic substances determines various effects on microalgae. Huminfeed^®^,(Humintech, Grevenbroich, Germany) an extract from leonardite, a highly oxidized lignite with high C: CH_2_ and C: H ratios, did not influence the growth or photosynthetic rate of the strain 61.81 *R. subcapitata* (synonym used *P. subcapitata*), strain 276-4d *Desmodesmus armatus,* or strain no. 2006 *Monoraphidium braunii* in a concentration of 2–20 mg C L^−1^. However, the chemistry of photosystem II is modified under treatment with Huminfeed^®^ (Humintech, Grevenbroich), as demonstrated by the modification of thermoluminescent light emission [82].

The humic lakes, developed primarily due to leaching of dissolved organic matter (DOM) from soil into continental water bodies, are natural aquatic ecosystems wherein the hormetic effects of humic substances influence the microalgae community structure [83]. DOM ranges from 0.5 to 100 mg C L^−1^ in the natural aquatic system [84]. For such concentrations, at the ecosystem level, the overlapping mechanisms of HS action on microalgae promote the development of species able to develop in multicellular structures, such as filamentous (Diatoms) or colonial (Cyanobacteria) structures [85], more resistant to oxidative stress than unicellular species [86].

## 3. Protective Effects of Humic Substances on Microalgae

**Figure 2:**
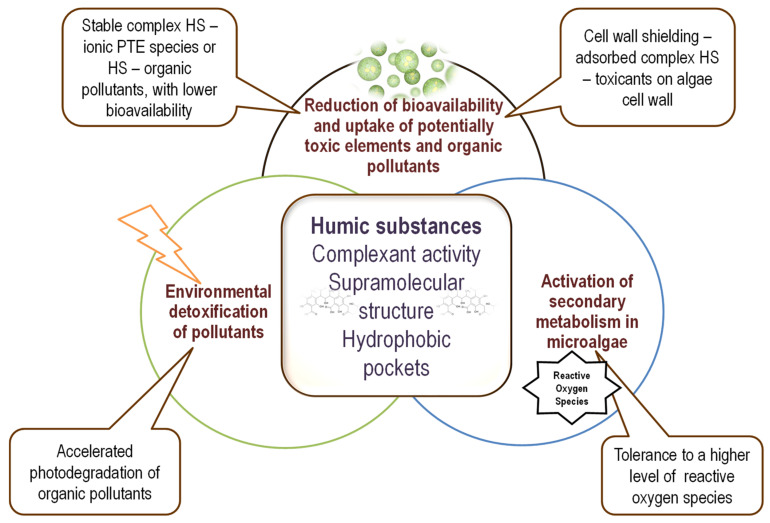
Illustration of the main mechanisms involved in microalgae protection by humic substances and the characteristics that are involved in these mechanisms.

Due to their specific properties, low concentrations of humic substances protect microalgae against the toxicity of potentially toxic elements and xenobiotics. The following main mechanisms are involved: (*i*) reduction of the bioavailability of potentially toxic elements and xenobiotics and modification of cellular uptake due to the formation of a protective coating of adsorbed HS-toxic complex on the microalgal cell wall; (*ii*) activation of photodegradation mechanism due to soluble HS–metal ion complexes; and (*iii*) higher tolerance to oxidative stress due to HS redox buffering activity and/or activation of secondary metabolism. These main mechanisms are presented in Figure 2.

Reduced cellular uptake due to the protective coating formed on the microalgae cell wall is the primary mechanism of action for protection against heavy metals and hydrophobic pollutants. The activation of photodegradation is usually induced by the HS–metal ion complex and is active on photosensitive pollutants. Increased tolerance to oxidative stress due to (bio)chemical priming mitigates the effects of compounds that induce reactive oxygen and nitrogen species at cellular levels. Illustrative examples of such mechanisms of protective HS effects on microalgae are presented in Table 2 and are further discussed.

Bioavailability is considered a key concept linking the changes in the concentrations of potentially toxic elements to their detrimental effects on biota [93]. Humic substances with low molecular weights (i.e., fulvic acids), soluble in water, increase the bioavailability of potentially toxic elements (PTE) in ionic forms and enhance the uptake of these ions by microalgae cells [94]. In the case of humic substances with supramolecular structures, i.e., humic acids, the bioavailability is decreased due to the adsorption of HA-ionic PTE on microalgae cell walls.

In the presence of a standard HA product (Suwannee River Humic Acid, SRHA), the Ag^+^ ion toxicity to microalgae *Chlamydomonas reinhardtii* and *R. subcapitata* (synonym used *P. subcapitata*) decreased, although the microalgae cells took up a higher amount of free silver ions. Most of the silver ions were bound to the cell walls and recovered in the cell debris fractions [95].

By increasing Cu ion addition to *C. vulgaris* culture, the microalgae registered evident growth inhibition and oxidative damage. The Cu-induced toxicity damage was alleviated when the culture was supplemented with HS, in an HS concentration-dependent manner [89]. Another study was performed to determine the influence of humic substances, applied at concentrations of 1 and 5 mg L^−1^, on the toxicity of Zn and Cd ions. The concentrations of metal ions were 390 µg L^−1^ for Zn^2+^ and 200 µg L^−1^ for Cd^2+^. The tested humic substances were Suwannee River fulvic acids and the humic acids extracted from peat and soil. HS and metal ions were tested in a microalgae photosynthesis inhibition assay using *R. subcapitata*. The effect was additionally studied by using a tangential flow ultrafiltration unit, which separated the colloidal HS from the dissolved HS. The humic acids significantly reduced the toxicity of metal ions. The results suggest that HS reduces Cd and Zn ions toxicity in two ways. The colloidal HS, i.e., the humic acids extracted from peat and soil, reduce the metal ions’ bioavailability because the formed complexes are relatively stable. The same high molecular weight supramolecular structures could adsorb onto algal surfaces and shield the cells from free Cd and Zn ions [87].

The shielding coat effect of the colloidal stable HS complex with metal ions that forms on the algal cell wall and acts as an inhibitor of subsequent adsorption of metal ions was demonstrated also for *Scenedesmus quadricauda* exposed to 100 μM Cd, Ni, Pb, and Hg ions for 24 h. The microalgae were cultivated for 30 days before their biomass was exposed to metal ions. The HS were tested at three concentrations of 1, 5, and 10 mg L^−1^. The authors pointed out that metals were mainly accumulated when no HS was added.

Another study investigated the effects of different irradiated HS against Pb^2+^ applied to freshwater microalga *Chlorella kesslerii*. The microalgae were exposed to 10^−6^ M Pb^2+^ applied as Pb(NO_3_)_2_ for 1 h in the presence of 10 mg C L^−1^ irradiated HS. Different commercial HS were studied: Suwannee River humic acid (SRHA), Suwannee River fulvic acid (SRFA), and Aldrich humic acid (AHA). The irradiation was performed with a solar simulator to mimic natural conditions. The experiment also intended to consider the irradiation factor and the effects of HS photoalteration on the bioavailability of toxic metals to microalgae. The results indicated that photoalteration of the tested HS decreased the amount of HS adsorbed to the microalgae cell wall. The adsorption of HS to algae is dependent on the HS composition and seems to be significant for HS with high hydrophobicity [88].

Microscale algal growth inhibition (μ-AGI) was developed in a high-throughput bioassay. This method was used to test the influence of humic substances on the toxicity of heavy metals (Hg, Cu) and hydrophobic organic pollutants (HOPs, such as pesticides and polycyclic aromatic hydrocarbons). The bioassay was performed on *C. reinhardtii*, strain C-239: UTEX-90, mt+. According to the International Humic Substances Society, several humic acid (HA) preparations were made and compared with two commercial preparations. Initially, the range of concentrations of HA was from 1 to 40 mg L^−1^. However, concentrations higher than 10 mg L^−1^ significantly inhibited microalgae growth. To mitigate the toxicity of heavy metals and HOPs, a concentration of 10 mg L^−1^ HA was used and proved to be effective. This μ-AGI method confirmed that the carboxylic acid content and molecular weight of HAs are essential for mitigating the toxic effects of Hg and Cu ions on *C. reinhardtii.* In the case of HOPs, mitigation of toxicity toward microalgae is directly related to the aromaticity and polarity of HAs [96].

The reduction in micro- and nano-sized plastic toxicity to microalgae by humic acids is also related to a mechanism involving a protective adsorbent coating that mitigates the adverse effects of micro/nano-plastic particles [90].

The humic acid–metal complexes are well-known for their role in environmental detoxification [97]. Detoxification of various aquatic pollutants reduces their toxicity on microalgae. One example is the photodegradation of glyphosate, a widely used herbicide, by the complex formed between HA–Fe^3+^. Glyphosate is highly toxic for the algae, inducing oxidative effects on the lipophobic intracellular environment of microalgae [98].

The experiments on enhanced photodegradation of glyphosate by complex compounds formed between humic acids and Fe^3+^ (HA-Fe^3+^) demonstrated that the optimum concentrations for the highest degradation rate were 20 mg L^−1^ HA and 0.5 mmol L^−1^ Fe^3+^ when the glyphosate concentration was 50 mg L^−1^ [99].

Humic acids reduce the toxicity of tetracycline toward microalgae. Tetracycline determined a dual response in the bioassay with *Coelastrella* sp. Tetracycline stimulated biomass and protein accumulation at concentrations lower than 2 mg L^−1^. Tetracycline concentrations higher than 2 mg L^−1^ reduced microalgae growth by more than 50%. Sodium salts of humic acids, at concentrations of 2 mg L^−1^ and 5 mg L^−1^, significantly reduced tetracycline toxicity. A high concentration of tetracycline induced high oxidative stress. Humic acid addition reduced oxidative damage and associated oxidative stress biomarkers, most probably due to (bio)chemical priming of secondary metabolism and the microalgae detoxification and defense systems [91].

Humic acids reduce the toxicity of graphene family materials (GFMs) toward microalgae, *Tetradesmus obliquus* (synonym used *Scenedesmus obliquus*) [100], and *Chlorella pyrenoidosa* [92]. The toxicity mechanism of GFMs is the membrane damage associated with oxidative stress [101]. Humic acids interact with GFMs and decrease GFM aggregation and adhesion to the microalgae cell membrane due to steric hindrance [92]. An additional protective effect is related to the reduced oxidative damage of GMFs in microalgae. Besides the (bio)chemical priming and activation of the tolerance against oxidative stress, another mechanism is related to the oscillating antioxidant–prooxidant characteristics of HS. The chinone–phenol moieties of HS have context-dependent redox characteristics [54]. In the “crowded” cellular environment with a high level of reactive oxygen species, HA exert antioxidant activity. The HA have a strong redox buffering activity due to the switch between oxidized quinone and reduced phenol [23,31].

The same effect of reducing the toxicity of graphene material, graphene oxide (GO), by humic acids due to the antioxidant activity was also reported for *C. vulgaris*, strain FACHB-8. GO also determines mutagenic effects (revealed by comet assay) due to the high level of induced reactive oxygen species. The concomitant HA applications reduced these mutagenic effects. At the same time, the nanoparticles formed by aggregation of the dissolved/suspended natural organic matter (NOM) acted as promotor of (geno) toxic GO effects [102].

## 4. Humic Substances as Microalgae Biostimulants

The scientific community has largely accepted the concept of plant biostimulants (PB) for more than a decade [103]. Plant biostimulants are a class of agrochemical inputs situated between fertilizers and plant protection products. The PB-specific effects are increased nutrient uptake and nutrient use efficiency, enhanced plant tolerance to abiotic stress, and improved edible crop quality [104]. Nowadays, plant biostimulants are classified as microbial and non-microbial plant biostimulants [105]. Non-microbial plant biostimulants are further classified into organic plant biostimulants and inorganic plant biostimulants [104]. Organic plant biostimulants are seaweed and botanical extracts [106,107,108,109], humic and fulvic acids [110,111], protein hydrolysates [112,113], chitosan [114,115], and other biopolymers [116,117,118]. Inorganic plant biostimulants are plant-beneficial elements, such as silicon [119,120] or selenium [121,122], which determine PB-specific biological effects when applied to cultivated plants.

The first generation of plant biostimulants was defined as a “formulated product of biological origin that improves plant productivity as a consequence of the novel or emergent properties of the complex of constituents” [53]. This definition was directly related to the difficulty of defining a mode of action specific and different from that of other agricultural inputs (fertilizers and plant protection products). Another obstacle was related to the difficulty in identifying plant biostimulant active ingredients. In the last two years, attempts have been made to define the pure organic active compounds from the main classes of plant biostimulants [123]. The HS effects on plants were considered the result of “chemical priming” [51].

The mirror concept of “microalgae biostimulant” is not often used in the scientific community, despite its utility. The main effect from plant biostimulants is the increased tolerance to abiotic stress, which is directly related to the molecular priming mode of action [124]. Enhanced tolerance to stress factors, especially in commercial-scale cultivation of microalgae and/or in mixotrophic conditions, could increase the overall productivity and profitability of microalgae cultivation [125].

The microalgae biostimulants can be divided into classes similar to those of plant biostimulants, non-microbial and microbial biostimulants [126]. Only a few studies refer to non-microbial algae biostimulants as a term used for describing the applied treatments based on the observed effects, most of them related to HS. Table 3 presents these papers and the effects of non-microbial biostimulants on different microalgae strains.

The growth-promoting effect is a direct result of the biostimulant effects. In the case of terrestrial plants, the growth-promoting term was one of the initial names given to biostimulant rhizobacteria [133], which are still in use today [134]. The non-microbial plant biostimulants were also considered ”plant growth promoters”—including humic acids [135]. The biostimulant effects are not limited to growth promotion. These effects are also related to the enhanced capacity of the treated photosynthetic organisms to adapt to the specific environmental conditions.

Phytohormones are among the most used microalgae growth-promoting products. Their benefits for utilization in microalgal biotechnology to increase microalgae growth and accumulation of metabolites of interest have been reviewed several times in recent years [136,137,138]. Recently, strigolactone analogs, phytohormones not detected in microalgae, which appear firstly in Charales [139], were demonstrated to promote microalgae growth and metabolite accumulation [140,141,142,143]. Besides phytohormones, HS and polyphenols (ferulic acid, protocatechuic acid, vanillic acid, phloroglucinol) were reported as non-microbial algae biostimulants/growth promotors. A commercial preparation of humic and fulvic acids was one of the ”biochemical stimulants” used to increase biomass productivity and metabolite content in *Chlorella sorokiniana* (UTEX 2805) [130]. Phloroglucinol promotes biomass accumulation and increases fucoxanthin synthesis in the microalga *Thalassiosira pseudonana* [144]. Phloroglucinol is also a plant tissue culture growth-promotor [145,146]. Methods to enhance the oil content of oleaginous microalgae based on HS were patented—e.g., on fulvic acid [147] or melatonin [148].

Most published papers refer to microbial (bacterial) microalgae growth promotors/biostimulants, recently reviewed in [149]. The microbial microalgae biostimulants, mainly bacterial strains, have been used for decades to improve mixotrophic microalgae cultivation [150,151]. Such bacterial strains were described as “microalgae growth-promoting bacteria” (MGPB) two decades ago, initially to describe the synthetic mutualistic interaction of *C. vulgaris* or *C. sorokiniana* with *Azospirillum brasilense* strain Cd [152,153]. A similar artificial consortium was established between oleaginous microalgae *Ankistrodesmus* sp. strain SP2-15 and *Rhizobium* strain 1011. The microalgal strain co-cultivated with bacteria was highly efficient in lipid production (up to 112 mg L^−1^ day^−^^1^ compared to 87 mg L^−1^ day^−1^ for the microalgae culture alone) and accumulation of omega-3 unsaturated fatty acids [154].

Bacteria from *Rhizobium* genera were proved to be associated naturally with the microalgae. Microalgae do not usually grow well in axenic conditions—they need a phycosphere colonized by associated/symbiotic bacteria for proper development [155].

Analysis of the diversity of phycosphere bacteria from various microalgae classes revealed that the majority of these bacteria are known to be plant growth-promoting bacteria (PGPB) [156]. The illustrative examples are bacteria from *Azospirillum* and *Rhizobium* genera [126]. Plant growth-promoting (Rhizo) bacteria were reconsidered as microbial plant biostimulants [157,158]. In similar manner, microalgae growth-promoting bacteria could be reconsidered as microbial microalgae biostimulants. Nevertheless, the significant difference between the “microalgae growth-promoting bacteria” and “microbial microalgae biostimulants” is related to the effects. The biostimulant activity is not related only to growth promotion—it involves increased tolerance to (abiotic) stress and enhanced accumulation of the ingredients of interest to cultivate the microalgae [159].

There are few reports regarding HS influence on microalgae co-cultivated with biostimulant bacteria. Fulvic acids from lignite leachate promoted the growth of *Desmodesmus subspicatus* (synonym used *Scenedesmus subspicatus*) co-cultivated with bacteria [160]. Humic-like substances from landfill leachate stimulated lipid accumulation on *C. pyrenoidosa* FACHB-9 co-cultivated in consortium with bacteria [161].

Due to their amphiphilic supramolecular structures with hydrophobic pockets [19], humic acids could influence the bioavailability of the chemical exo-signals used for communication inside the microalgae microenvironment: microalgae to microalgae, microalgae to associated bacteria, bacteria to bacteria. Such exo-signals are, in general, hydrophobic compounds, e.g., acyl-homoserine-lactone (AHL), used for inter-specific quorum sensing (QS, detecting bacterial density from the same ecotype) in Gram-negative bacteria [162]. The importance of QS signals for the microalgae–bacteria interactions was recently reviewed [163].

The gap in knowledge regarding interactions between humic substances and quorum sensing molecules is significant. The data are scarce and exist only for the soil environment and wastewater anammox anaerobic treatment. Water-soluble humic substances repress the formation of QS signals in *Sinorhizobium meliloti* and promote the efficiency of symbiotic nitrogen fixation in *Medicago sativa* [164]. Fulvic acid stimulated AHL release in the anammox (anaerobic ammonium oxidation) and improved bacterial activity [165]. Further studies are needed to determine the effects of HS on QS and the underlying mechanisms, especially in microbial consortia involving microalgae. It will help to effectively utilize HS as biostimulants for microalgae cultivated in xenic conditions when large populations of associated phycosphere bacteria producing QS signals are present. HS could modulate the bioavailability of QS signals similarly to cyclodextrin—inclusion inside a hydrophobic pocket [166].

There are no data regarding the interactions between HS and the various other infochemicals present in the microalgae culture. For example, the microalgae from the *Scenedesmaceae* family respond to grazers natural cues by forming flocculating colonies [167]. Such mechanisms are of interest for microalgal biotechnology as a lower-cost harvesting process [168]. HS could support the initial formation of the extracellular matrix of polysaccharides and lipids required for colony formation and flocculation [169], reducing the metabolic costs of colony formation [170]. The surfactant agents, such as linear alkyl benzene (LAB) [171], benzalkonium bromide (BZK) [172], sodium dodecyl sulfate (SDS) [172,173], and nonionic surfactant polyoxyethylene (40) nonylphenol ether (NPE) [172] enhance microalgae colony formation in the presence of smaller amounts of grazers natural cues or even in their absence [171]. Such effect should be considered in terms of ecotoxicological risk assessment, but also for its biotechnological potential related to microalgae harvesting. HS, due to their complex structure that generates other specific chemical features and associated biological activity, could be modulator of the final inducing step of colony formation in microalgae biotechnology.

The utilization of humic substances as microalgae biostimulants benefits from the advances in knowledge related to HS structure–activity. Despite HS complexity, the various analytical techniques, combined with chemoinformatic tools, allow an accurate estimate of HS biological activity based on chemical and chemo-physical features. The ratio between HA electron-accepting capacity (EAC) and electron-donating capacity (EDC) that is related to quinone and semi-quinone and, respectively, to polyphenols and glycosylated polyphenols, predicted the plant biostimulant activity of HA in corn seedlings [174]. The low molecular weight HS, which penetrate the cell membrane more easily, modulate intracellular signals. The high molecular weight HS interact with the cell membrane receptors [175]. The ability of HS to act as eustressors (positive stressors) on rice plants is related to aromaticity, hydrophobicity, aliphaticity, and polarity [176]. A combination of different HS, of different origins, with different and well-characterized hydrophobicity and hydrophilicity, produced bioactive and environmentally friendly products [177].

The projection of the latent structure (PLS) regression, using molecular structures obtained by ^31^P-NMR spectra of derivatized samples and ^13^C-CPMAS-NMR spectra obtained directly on samples, predicted the biological activity of humic-like biostimulants obtained from bioeconomy side-streams [178]. Because the HS structure–activity relationship is not linear, the linear artificial neural network (ANN) based on Fourier-transform infrared (FT-IR) spectroscopy predicted better the biological activity of HS extracted from peat [176]. Fourier transform ion cyclotron resonance mass spectrometry (FT-ICR MS) was used to differentiate the molecular signature of humic acids from different sources. The degree of humification, determined according to aromaticity and degree of saturation, increases the HA from soil, river, and leonardite/oxidized lignite [179]. FT-ICR MS, in combination with ^13^C-CPMAS NMR spectroscopy and FT-IR ATR, revealed the co-existence of anti-oxidant and pro-oxidant moieties and properties of humic acid extracted from lignite [180]. These complex chemical properties were related to the biostimulant effects on tomatoes cultivated under nutritional stress [180].

Several constraints should be considered in using HS as biostimulant for microalgae. HS have higher electron-donating capacities (EDC) in the aquatic environment than in soil [181]. The physiological window of beneficial HS doses is narrow, and inhibitory doses must be avoided. Microalgae release into their cultivation media various organic compounds with various molecular masses, including aromatic amino acids such as tryptophan and tyrosine, heterocyclic pigments such as biopterin, proteins, etc., that generate humic-like substances, algal organic matter (AOM) [182]. This dissolved organic matter that results from microalgae organisms is also called humic matter due to its aromaticity (π-π systems) related to significant fluorescence [183,184]. In several cases, accumulations of this organic matter during microalgae cultivation block water reuse. Humic acids produced from AOM are the major microalgae growth inhibitors for *E. gracilis* strain CCAP 1224/5Z [185], *S. acuminatus* strain GT-2 [186], and *N. oceanica* LARB-202-3 [187]. Filtration of the spent media through activated charcoal and oxidative degradation by ultraviolet light or ozonization effectively reduces the toxicity of the algogenic HS (organic matter—AOM) to the microalgae [185,186,187]. Similar treatments could be used in the situation of enhanced production of AOM under HS treatment or in the situation of biostimulant HS accumulation.

Overall, HS fulfill the criteria required for non-microbial microalgae biostimulants. HS modulate microalgae growth and mineral nutrient availability. HS increase tolerance to chemical stressors and enhance the accumulation of microalgae metabolites. These effects are proposed to result from the “chemical priming”, i.e., a “preparedness” condition that promotes faster metabolic pathway activation. Similar evidences supporting the hypothesis of “chemical priming” in terrestrial plants by HS [51] were already presented in Section 2 for HS–microalgae interaction.

## 5. Humic Substance Interactions with Microalgae Harvesting by Flocculation

Interest in microalgae cultivation and the resulting biomass utilization has increased significantly in the last two decades. The main driving interest was initially related to the CO_2_ mitigation potential combined with biofuel production [188]. The fast growth rate and the high lipid content of oleaginous microalgae represented arguments to consider microalgae as an alternative solution to fossil fuels [189]. Integration with wastewater treatment was another benefit of the microalgae-based fuels [190]. However, several problems of microalgae cultivation have prevented the development of microalgae-based biotechnologies, especially those focused on the production of bulk chemicals/biofuels [189,191,192]. One of these issues related to the profitability of the microalgae-based product is the dewatering/harvesting step [191,192,193].

Efficient harvesting and dewatering represent a major bottleneck for all microalgae-based biotechnologies, including those focused on more profitable products, such as carotenoids [194]. The dilute nature of microalgae in suspension requires high energy consumption for this initial step in microalgae processing [195]. Microalgae flocculation from growing media was proposed to be a solution to reduce energy consumption for harvesting and dewatering [196]. However, the costs of flocculants reduce the profitability of this solution [195]. Affordable and natural flocculants present several advantages in terms of costs and environmental impacts [197,198,199].

The utilization of HA as a flocculant for microalgae exploit their capacity to bind to the microalgae cell walls and/or form a network with other microalgae flocculating agents. HA were used as flocculant together with cationic aminoclay nanoparticles. A patented composition of aminoclay (AC) and HA with 0.1–0.3 g L^−1^ HA and 3–7 g L^−1^ AC content was used to harvest oleaginous microalgae—*Ankistrodesmus* sp., *Anacystis nidulans*, *Biddulphia aurita* [200]. In cooperation with scientists from other Korean research entities, the patent authors published a short communication demonstrating that AC-HA forms a network that captures oleaginous *Chlorella* sp. microalgae [201].

Flocculation of microalgae biomass was enhanced by the utilization of the humic-like exopolymers produced by *S. acuminatus*. When the humic-like exopolymers were used, the Al^3+^ ionic coagulant concentration was almost 20 times reduced (from 77.6 to 4.5 mg L^−1^) [202]. In other situations, the HS presence reduces the harvesting microalgae efficiency by flocculation process. HS reduces the efficiency of *T. obliquus* (synonym used *S. obliquus)* FSP-3 flocculation by ozone [203] or *C. vulgaris* 211–11b flocculation by calcium phosphate precipitation [204]. Therefore, the biotechnological process aiming to utilize HS as microalgae biostimulants should also consider the HS interference with microalgae flocculation.

## 6. Enhanced Biotechnological Production of Microalgae-Based High-Value Products by Humic Substances

Nowadays, the interest in microalgae cultivation is also related to the products with high added value that can be produced by microalgal biotechnology—Table 4.

The potential use of humic substances as microalgae biostimulants must take into account the mixotrophic cultivation of microalgae (since parts of the HS could be used as C or N sources by microalgae and/or symbiotic associative bacteria) and to the biosynthesis of the products that are not affected by the presence of HS (since the separation of HS in the downstream process is complicated and increases production costs). The mixotrophic process for microalgae cultivation, which leads to higher biomass concentrations compared to auxotrophic grown microalgae, and the acceleration of microalgae growth (including by the use of microalgae biostimulants) are solutions that reduce the energy costs for microalgae cultivation and increase the profitability of microalgae cultivation on a large scale [124].

The products with high added value that can be obtained from microalgae have a plethora of applications within the biomedical, food, feed, and agriculture sectors. The investigation of the effects of HS on the production of these products is still in its infancy but could have a significant impact. The few studies available focused on certain compounds such as lipids, fatty acids, and carbohydrates. Still, other products such as carotenoids, polyphenols, polyamines, and proteins should be investigated more in-depth. The humic acids, applied in concentrations between 0.1 and 0.5 mg L^−1^, were proven to stimulate the growth of microalgae *Dunaliella salina* and *Nannochloropsis salina* used in aquaculture for fish feed. The accumulation of chlorophyll *a*, carotenoids, lipids, and proteins was also stimulated by humic acids [205]. Other preliminary studies involving fulvic acid and astaxanthin [206] are promising.

Moreover, because HS were shown to be safe for many applications, there would be no need for further purification or separation. In some cases, HS were shown to have positive effects and, therefore, could even act synergistically with products from microalgae. HS were found to be antimicrobial and have prebiotic, anti-inflammatory, antioxidant, and immunomodulating activities in vertebrates [18]. HS were proven to have health-promoting effects on fish—reducing stress and fungal disease and stimulating probiotic bacteria [207]. Therefore, their use to stimulate the production of high-added-value feed ingredients (e.g., astaxanthin) is highly feasible. Trace HS will not negatively influence the value of the feeds. Humic acids and yeast-derived immunomodulator glucan were shown to have synergistic stimulation effects on the immune system [208]. These findings suggest that this synergism could apply also to similar bioactive compounds from microalgae, these vast possibilities being unexplored at the moment.

The production of plant biostimulants from microalgae biomass is one of the most recent developments in microalgal biotechnology [209]. Seaweed extracts represent a well-known and highly effective class of plant biostimulants [106]. However, the microalgae biomass is more affordable and less complicated to standardize as a raw material for plant biostimulants [210]. The sustainability of microalgae-based plant biostimulant production is higher than that of seaweed-based plant biostimulants [211].

A pioneering work of our group demonstrated the plant biostimulant effect of microalgae extracts resulting from the biomass of microalgae *Nannochloris* sp. 424-1, CCAP 251/10, cultivated mixotrophically [212].

Polyamines represent another category of endo- and exo-signals that have protectant action against abiotic stress. The *Arthrospira* (synonym used *Spirulina*) *platensis* biomass submitted to enzymatic hydrolysis for four hours generates hydrolysate with high spermine content that biostimulates field-grown lettuce [213].

The (exo)polysaccharides are other compounds from microalgae that contribute to the specific plant biostimulant action due to the activation of plant innate immunity. Their activity is related to the in situ formation of oligosaccharins [214]. Oligosaccharins are endo-signals that regulate plant growth, development, and gene expression, from the category of plant innate immunity elicitors, i.e., pathogen-associated molecular patterns (PAMPs); microbe-associated molecular patterns (MAMPs); and damage-associated molecular patterns—DAMPs [215].

The polysaccharides extracted from eukaryotic and prokaryotic microalgae (*D. salina* strain MS002, *Porphyridium* sp. strain MS081, *D. salina* strain MS067, *Phaeodactylum tricornutum* strain MS023, *Desmodesmus* sp., *A. platensis* strain MS001) and applied as leaf treatment induced the biochemical markers related to the activation of the defense pathways in tomatoes—chitinase, 1,3 beta-glucanase, phenylalanine ammonia-lyase (PAL), and peroxidase—POX [216].

The crude extract containing polysaccharides from the Moroccan strain of *C. vulgaris* and *C. sorokiniana* injected in 40-day old tomato plants determined an increase in β-1,3-glucanase activity and significantly increased the content of polyunsaturated fatty acids. The exo-polysaccharides extracted from the halophytic microalgae *D. salina* MS002, cultivated in hypersaline media, enhance tolerance of *Solanum lycopersicum* var. Jana F1 to salt stress [217].

Protein hydrolysate is another source of plant biostimulants from microalgae. The bioactive peptides resulting from enzymatically hydrolyzed microalgae proteins sustain plant hormone biosynthesis in the plant tissue, activate primary metabolism, and stimulate nutrient uptake [108]. The amino acids from microalgae protein hydrolysate, especially the glutamic and aspartic acids, modulate primary metabolism and increase nutrient use efficiency due to anaplerotic reactions of the tricarboxylic acid cycle [218]. The de-oiled microalgae biomass, resulting from the production of third-generation biodiesel, is an effective hydrolysate source that promotes plant growth and development [219]. Table 5 summarizes the active ingredients in microalgae extracts that contribute to the plant biostimulant effect.

**Figure 3:**
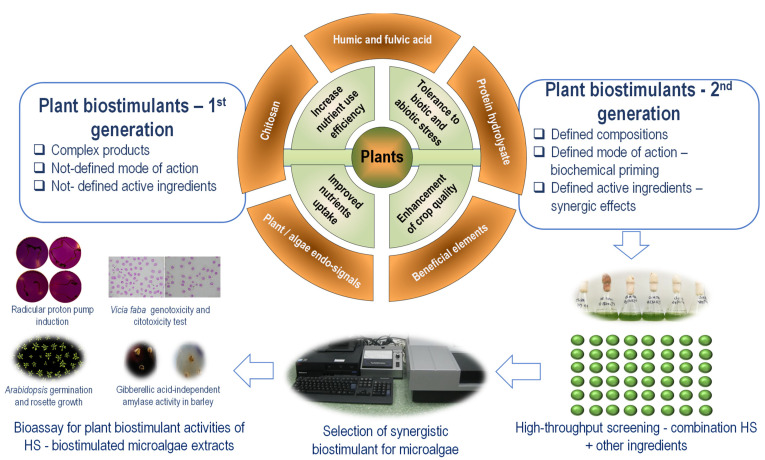
Development of second-generation plant biostimulants based on microalgae bioassay of interactions between humic substances and other active ingredients. The high-throughput screening on microalgae selects a combination with synergic effects that are further verified in plant biostimulant bioassay. Extracts of HS-biostimulated microalgae could be also used in association with the added HS for their activity as plant biostimulants, by using several bioassays—radicular proton pump induction [220], *Vicia faba* genotoxicity and cytotoxicity test [221], *Arabidopsis* germination and rosette growth [222], Gibberellic acid-independent amylase activity in barley [223].

HS components could act on terrestrial plants complementarily with the active ingredients from microalgae extract. The hormetic HS effect on microalgae could support the development of the HS-based second generation of plant biostimulants (Figure 3).

The high-throughput screening bioassay proposed in Figure 3 is multifunctional. It could be used to select a synergic combination of HS with other PB-active ingredients. Additionally, it could be used to generate PB based on HS-biostimulated microalgae, selecting the optimum HS concentrations active in microalgae and further synergizing microalgae extracts’ active ingredients. A high-throughput screening assay based on microalgae application of HS and other active ingredients could select an optimal ratio between components. The hormetic effect allows fine-tuning of the optimal combination between HS and other active ingredients of plant biostimulants, including extract of microalgae. The selected combination can be further tested in a battery of plant bioassays to identify the synergistic plant biostimulant compositions. The compatibility between HS and microalgae was already proven. A combination of commercial PB based on HS (humic acids extracted from leonardite, 33% C) and lyophilized microalgae biomass *D. subspicatus* (synonym used *S. subspicatus*) in aqueous suspension has been shown to exert synergic plant biostimulant effects on onions grown in an organic farming system [174]. Therefore, microalgae stimulation by in situ application of humic substances, at a concentration from the initial range of hormetic effect, could generate a second-generation plant biostimulant. These plant biostimulants from the second generation are products based on synergic combinations of active ingredients [224].

**Figure 4:**
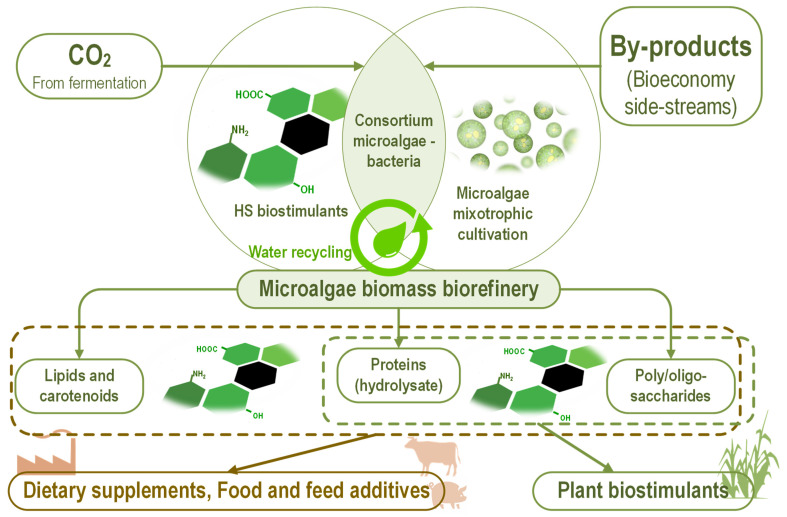
Using the humic substances as biostimulants for microalgae in integrated biotechnology, converting by-products from bioeconomy and carbon dioxide from fermentation process into high-value bioproducts—dietary supplements, food and feed additives, plant biostimulants. The complementary or even synergic HS interactions with components from microalgae used as active ingredients in these bioproducts underpin HS utilization as a biostimulant for microalgae.

Using humic substances as biostimulants for microalgae cultivation could be achieved in an integrated/zero-waste microalgae-based biotechnological process (Figure 4).

HS promote microalgal metabolite accumulation under different stress conditions. Fulvic acids induce the accumulation of lipids in nitrogen starvation conditions in *Monoraphidium* sp. FXY-10 [225]. Melatonin and fulvic acid enhance lipid and photosynthetic pigments accumulation in *Heveochlorella* sp. Yu MK829186.1 due to modulation of nitrogen and reactive oxygen species [226]. Fulvic acids promote astaxanthin accumulation in *H. pluvialis* KM115647 under high light and nutrient starvation stress [206].

HS complement/synergize microalgae components used as active ingredients for dietary supplements or plant biostimulants in several ways. Humic acid increased water solubility and photostability of β-carotene [227]. Humic acids interact in a synergic manner with β-glucan for activation of the immune system [208], protection against liver injury induced by chemical agents [228], and binding aflatoxin B_1_ in vitro [229].

## 7. Conclusions

Humic substances (HS) are supramolecular structures stabilized by hydrophobic interactions. In aqueous solutions/suspensions, HS generates a more dynamic supramolecular structure. Due to the dynamic structure of HS in solution/suspension, the main HS physicochemical characteristics are enhanced. The biological activities related to these characteristics are also enhanced. Amplified HS biological activities in water systems and the unicellular nature of microalgae make more evident the hormetic effects of HS on aquatic photosynthetic microorganisms.

Overall, HS fulfill the criteria required for a non-microbial microalgae biostimulant. HS increase mineral nutrient availability, microalgae growing rate, and biomass accumulation. HS increase tolerance to chemical stressors and enhance the accumulation of ingredients of interest for microalgae cultivation under abiotic stress conditions.

The HS biostimulant effect on microalgae could be exploited to improve the yield of high added-value products obtained by microalgae cultivation, such as food and feed additives, dietary supplements, and plant biostimulants.

## Figures and Tables

**Table 1:** Dual response of microalgae to humic substances according to the dose.

Humic Substances	Tested Microalgae	Concentration–Effect	Reference
Humic acid extracted from lignite	*Scenedesmus acutus* Meyen Tomaselli 8 *Chlorella vulgaris* C-3 *Anabaena variabilis* 786 *Nostoc commune*	Up to 10 mg L^−1^ enhances biomass accumulation 100 mg L^−1^ enhances protein accumulation 1 g L^−1^—inhibition	Pouneva, 2005 [64]
Humic acids extracted from lake sediments	*Desmodesmus communis* 41.71 *Chroococcus minutus* 276-4b	0.3 mg L^−1^ increases the number of *D. communis* cells Inhibitory effects on *C. minutus*	Prokhotskaya and Steinberg, 2007 [63]
Humic acid extracted from lignite, Artificial humic acid	*Raphidocelis subcapitata* 61.81 *Monoraphidium braunii* 2006 *Synechocystis* PCC 6803 *Microcystis* *aeruginosa* PCC 7806	0.17 mM stimulates photosynthesis 4.7 mM reduces cell development and inhibits photosynthesis	Bährs et al., 2012 [62]
Humic acids extracted from soils	*Chlorella vulgaris* co. 157	0.01–0.03% activation >0.03% inhibition	Toropkina et al., 2017 [61]
Commercial (Suwannee River) humic acids	*Scenedesmus capricornus* FACHB-271 *Chlorella* spp. FACHB-271	0.05–0.1 mg L^−1^ stimulation 1.0 mg L^−1^ inhibition	Zheng et al., 2022 [60]

**Table 2:** Examples of protective effects of humic substances (HS) against the ionic forms of potentially toxic elements and xenobiotics/chemical pollutants.

Aquatic Pollutant	Tested Microalgae	Main Mechanism	Reference
Cd^2+^, Zn^2+^	*Raphidocelis subcapitata*	Supramolecular structure adsorbed in the cell wall surface, which complexes toxic ions	Koukal et al., 2003 [87]
Pb^2+^	*Chlorella* *kesslerii*	HS–Pb^2+^ complexes are adsorbed on microalgae cell walls. HS photoalteration reduce the adsorption of HS–Pb^2+^ to microalgae surface	Spierings et al., 2011 [88]
Cu^2+^	*Chlorella* *vulgaris*	HS addition reduces bioavailability of Cu^2+^ and decreases the secretion of exopolysaccharide matrix involved in Cu^2+^ toxicity	Shi et al., 2021 [89]
Microplastics	*Chlorella* *vulgaris*	HA decrease electrostatic interactions between polystyrene nanoplastics and microalgae and ameliorate cellular aggregation	Hanachi et al., 2022 [90]
Tetracycline	*Coelastrella* sp.	Reduction of oxidative stress damage (due to biochemical priming)	Tong et al., 2020 [91]
Graphene family materials (GFMs)	*Chlorella* *pyrenoidosa*	Reduction of absorption due to steric hindrance HA- GFMs Reduction of oxidative stress damage	Zhao et al., 2019 [92]

**Table 3:** Effects of non-microbial biostimulants on different microalgae strains.

Compound	Tested Microalgae	Main Effects	Reference
Humic-like extract of anaerobic digestate (D-HL) Humic-like extract of residues from rapeseed oil production (B-HL) Humic-like extract of tomato plants (T-HL)	*Chlorella* *vulgaris* CCAP 211/11C *Scenedesmus* *quadricauda*	Increased biomass production (~25–40%) by DH-L and TH-L Increased oil accumulation (~60–90%) by DH-L and TH-L Increased unsaturated fatty acid content by B-HL Increased carbohydrate content by B-HL	Puglisi et al., 2018 [56]
Fulvic acid	*Haematococcus pluvialis* KM115647	Increased astaxanthin and lipid content	Zhao et al., 2019 [127]
Selenium and betaine	*Dunaliella* *salina*	Increased carotenoid and antioxidant activity	Constantinescu-Aruxandei et al., 2019 [128]
Humic acids	*Euglena* *pisciformis* AEW501	Increased biomass yield Higher lipid content Higher content of unsaturated fatty acids	Fan et al., 2022 [129]
Humic and fulvic acid (commercial preparation)	*Chlorella* *sorokiana UTEX2805*	Increased biomass yield Increased metabolite accumulation	Hunt et al., 2010 [130]
Lignosulfonate	*Euglena gracilis* NIES-48	Increased biomass yield Higher lipid content	Zhu and Wakisaka, 2021 [131]
Phenolic precursors of lignin			Zhu et al., 2021 [132]

**Table 4:** Market potential of microalgae-based high-value compounds. Reconstructed and updated, from Velea et al., 2017 [125].

High Value-Added Compounds	Market Estimation		Price Range (USD kg^−1^)
	Estimated Value (mio.US$)	Compound Annual Growth Rate—CAGR	
Plant biostimulants	3200 (2021) ^a^	12.1% (2021–2026)	60–90 ^a^
Carotenoids (total)	1500 (2019) ^b^	4.2% (2019–2027) ^b^	-
Beta-carotene	532 (2019) ^a^	3.3% (2014–2019) ^b^	300–1500 ^b^
Lutein	314 (2019) ^a^	3.6% (2014–2019) ^b^	-
Astaxanthin	423 (2019) ^a^	2.3% (2014–2019) ^b^	200–7000 ^b^
Canthaxanthin	117 (2019) ^a^	3.7% (2014–2019) ^b^	100–500 ^d^
Omega-3 fatty acids	2100 (2020) ^c^	7.4% (2020–2028) ^c^	80–160 ^d^

^a^—https://www.marketsandmarkets.com/Market-Reports/biostimulant-market-1081.html, accessed on 20 April 2022. ^b^—https://www.marketsandmarkets.com/Market-Reports/carotenoid-market-158421566.html, accessed on 20 April 2022. ^c^—https://www.grandviewresearch.com/industry-analysis/omega-3-market, accessed on 20 April 2022. ^d^—Borowitzka, 2013 [205].

**Table 5:** Active ingredients from microalgae-based microbial biostimulants.

Active Ingredients	Microalgae	Main Mechanism	Reference
Polysaccharides	*Dunaliella salina* MS002, *Porphyridium* sp. MS081, *D. salina strain* MS067, *Phaeodactylum tricornutum* MS023, *Desmodesmus* sp., *Arthrospira. platensis* MS001	Elicitation of the plant defense mechanisms and activation of secondary metabolism	Rachidi et al., 2021 [116]
Osmoprotectants—glycine-betaine and proline	*Nannochloris* sp. 424-1, CCAP 251/10	Protection of plants against hydric stress, enhanced water use efficiency	Oancea et al., 2013 [214]
Osmoprotectants—polyamines	*A. platensis*	Increased biomass yield Higher lipid content Higher content of unsaturated fatty acids	Mógor et al., 2018 [215]
Protein hydrolysate	*Chlorella vulgaris*	Activation of primary metabolism, Increased nutrient uptake and nutrient use efficiency	Maurya et al., 2016 [220]

## Data Availability

Not applicable.
